# Revision of *Amana yunjuensis* (Liliaceae) based on morphological and molecular evidence: synonym of *Amana anhuiensis*

**DOI:** 10.3897/phytokeys.269.181258

**Published:** 2026-01-23

**Authors:** Meizhen Wang, Shenlu Zhang, Liang Guo, Qing Ma, Pan Li

**Affiliations:** 1 Keqiao Science and Technology Innovation Center, Zhejiang Shuren University, Hangzhou, Shaoxing 312028, China College of Biology and Environmental Engineering, Zhejiang Shuren University Hangzhou China; 2 College of Biology and Environmental Engineering, Zhejiang Shuren University, Hangzhou 310015, China Keqiao Science and Technology Innovation Center, Zhejiang Shuren University Hangzhou China; 3 Agricultural and Rural Bureau of Lin‘an District, Hangzhou 311300, China Agricultural and Rural Bureau of Lin‘an District Hangzhou China; 4 Key Laboratory of Biodiversity and Environment on the Qinghai-Tibetan Plateau, Ministry of Education, School of Ecology and Environment, Xizang University, Lhasa 850000, China Xizang University Lhasa China; 5 Motuo Biodiversity Observation and Research Station of Xizang Autonomous Region, Motuo 860700, China Motuo Biodiversity Observation and Research Station of Xizang Autonomous Region Motuo China

**Keywords:** *Amana anhuiensis*, *Amana yunjuensis*, synonym, taxonomic revision

## Abstract

Based on the integrated morphological and molecular evidence, *Amana yunjuensis*, originally described from Mt. Yunju in Jiangxi province, is demonstrated not essentially different from *A. anhuiensis*. Indistinguishable diagnostic characters, nested phylogenomic relationships, and no genetic distinctiveness of population genomic structure all indicate that *A. yunjuensis* does not represent an independent evolutionary lineage. Accordingly, *A. yunjuensis* should be treated as a taxonomic synonymy of *A. anhuiensis*.

## Introduction

*Amana* Honda (1935: 20) is endemic to East Asia (the eastern and central part of China, Japan and the Korean Peninsula) and composed of 12 species: *A. edulis* (Miq.) Honda (1935: 20), *A. erythronioides* (Baker) D.Y.Tan & D.Y.Hong (2007: 441), *A. anhuiensis* (X.S. Shen) D.Y.Tan & D.Y.Hong (2008: 394), *A. kuocangshanica* D.Y.Tan & D.Y.Hong (2007: 437), *A. wanzhensis* L.Q.Huang, B.X.Han & K.Zhang (2014: 120), *A. baohuaensis* B.X.Han, Long Wang ter & G.Y.Lu (2019: 45), *A. latifolia* (Makino) Honda (1935: 21), *A. nanyueensis* P.Li & L.X.Liu (2023: 65), *A. tianmuensis* P.Li & M.Zhen Wang (2023: 66), *A. hejiaqingii* M.Zhen Wang & P.Li (2022: 287), *A. yunmengensis* P.Li, M.Zhen Wang & Zi Yi Wang (2023: 18) and *A. polymorpha* M.Zhen Wang & P.Li (2024: 151) (Ohwi and Kitagawa 1992; Chen and Mordak 2000; Shen 2001; Tan et al. 2007, 2008; Han et al. 2014; Wang et al. 2019; Wang et al. 2022; Wang et al. 2023a; Wang et al. 2023b; Wang et al. 2024). Nearly half of these species were newly discovered and published in recent years. The morphology of *Amana* is relatively simple, and interspecific difference is mainly reflected in characters such as the density of hair inside the bulb tunic, leaf shape and vein, bract type and number, and the colour of flowers and anthers (Wang et al. 2024). Besides, during our comprehensive and systematic field surveys, some individuals with atypical traits were occasionally found, for example, *Amana edulis*, with three bracts and one white vein. Therefore, species identification in *Amana* cannot rely solely on morphological traits, which correspond only to the morphological species concept (Wilkins 2009). Instead, multiple lines of evidence are required to corroborate taxonomic boundaries with confidence, namely integrative taxonomy (de Queiroz 2007; Liu 2016; Hong 2020).

In 2024, we noted the publication of a new species *Amana yunjuensis* B.X.Han & X.W.Song (2024: 232) (Song et al. 2024). However, based on comprehensive field investigations and molecular data analyses (Wang et al. 2024; Liang et al. 2024), we conclude that this *Amana* population distributed in Mt. Yunju should, in fact, be referred to as *Amana anhuiensis*. We therefore propose the following taxonomic revision.

## Material and methods

### Sampling and sequencing

In addition to the data previously reported by Wang et al. (2024), sampled populations from Mt. Tianzhu in Anhui Province, Mt. Yunju and Mt. Lushan in Jiangxi Province, and Tuanfeng County in Hubei Province were studied through morphology and molecular analyses (Table 1). Fresh leaf tissues were immediately frozen in liquid nitrogen and subsequently stored at –80 °C. RNA libraries were prepared using the NEBNext Ultra RNA Library Prep Kit for Illumina (NEB, USA) according to the manufacturer’s protocol, and sequencing was performed on an Illumina NovaSeq 6000 platform, generating 150-bp paired-end reads (Novogene, Tianjin, China).

**Table 1.** Information of collected samples of *Amana anhuiensis* and *A. yunjuensis*.

Species	Collect number	GPS coordinates	Altitude/m	Location
*Amana anhuiensis*	LJK61	30.7235°N, 116.4537°E	1711	Mt. Tianzhu, Qianshan County, Anhui Province
	LJK62	30.7235°N, 116.4537°E	1711	Mt. Tianzhu, Qianshan County, Anhui Province
	CMQ2015075	30.7410°N, 116.4526°E	1207	Mt. Tianzhu, Qianshan County, Anhui Province
	WMZ1480	30.7235°N, 116.4537°E	1711	Mt. Tianzhu, Qianshan County, Anhui Province
	WMZ1750	30.8623°N, 115.0971°E	789	Tuanfeng County, Hubei Province
	WMZ1755	29.5553°N, 115.9719°E	616	Mt. Lu, Lushan City, Jiangxi Province
*Amana yunjuensis*	LJK63	29.0967°N, 115.5767°E	703	Mt. Yunju, Yongxiu County, Jiangxi Province
	LP173014	29.0968°N, 115.5767°E	703	Mt. Yunju, Yongxiu County, Jiangxi Province
	WMZ1499	29.0968°N, 115.5768°E	711	Mt. Yunju, Yongxiu County, Jiangxi Province

### Morphological analysis

The morphological characteristics of bulbs, bracts, leaves, flowers and fruits were measured and recorded. Principal coordinates analysis was conducted based on 15 traits with R package vegan v.2.8.0 (Oksanen et al. 2025) and ggplot2 v.3.3.6 (Wickham 2016), including inner side of bulb tunic texture (thinly papery: 0; papery:1), bulb tunic inside (glabrous: 0; sparsely villous: 1; densely villous-woolly: 2), length and width of lower leaf, lower leaf length/width, length and width of upper leaf, upper leaf length/width, length from the apex to the widest part of the lower and upper leaf, number of bract, bract length and width, number of flower and colour of anther (yellow: 0; purple: 1). A total of 21 individuals (13 of *A. anhuiensis* from two populations and eight of *A. yunjuensis* from two populations) were used for analysis (Suppl. material 1).

To assess morphological differences in bulb traits between *Amana anhuiensis* and *A. yunjuensis*, the bulb diameter and bulb height of each individual were measured using a vernier caliper to the nearest 0.01 mm (Suppl. material 2). For each specimen, a bulb size index was calculated as the product of bulb diameter and height (diameter × height) to approximate overall bulb volume. Statistical comparisons of the three variables (diameter, height and size index) between species were performed using the non-parametric Wilcoxon rank-sum test (Wilcoxon 1945), as the data did not conform to normal distribution. Boxplots were generated in R (v4.5.0) using the ggplot2 and patchwork packages (Pedersen 2024) to visualize the distributions, and p-values were displayed above each comparison.

### Phylogenetic analyses

Transcripts were assembled using Trinity v2.14.0 (Grabherr et al. 2011) with default parameters, and putative protein-coding sequences were predicted using TransDecoder v5.5.0 (Haas et al. 2013). Redundant transcripts were removed with CD-HIT v4.8.1 (Li and Godzik 2006) using the parameters ‘-c 0.95 -n 8’. Orthologous groups were identified across all samples (12 species and 101 individuals) using OrthoFinder (Emms and Kelly 2019), and gene clusters present in all individuals with a maximum copy number of fewer than three were retained, resulting in 2,567 protein sequences. Orthologs were inferred following the tree-based orthology strategy proposed by Yang and Smith (2014). Gene trees were reconstructed using RAxML v8.2.12 (Stamatakis 2014), and long terminal branches (more than 0.5 or 10 times longer than their sister lineages) as well as deep paralogs were pruned. The Monophyletic Outgroups (MO) approach (Yang and Smith 2014) was applied to infer orthologs, with the minimum taxon occupancy set to 60, resulting in 1,652 monophyletic trees. Maximum likelihood (ML) analyses were conducted in IQ-TREE 2 (Minh et al. 2020) using 1,000 bootstrap replicates, and the best-fitting models for each gene partition were selected with ModelFinder (Kalyaanamoorthy et al. 2017). The final phylogenetic trees were visualized and exported using FigTree v1.4.4 (http://tree.bio.ed.ac.uk/software/figtree/).

### Admixture analysis

Orthologous genes shared by at least 75% of individuals across the 12 *Amana* species were merged and used as a reference dataset. In total, 13,023 genes were obtained, and the longest transcript of each gene was retained to represent its sequence. Clean reads were aligned to the reference using BWA-MEM v0.7.17 (Li 2013) and sorted with SAMtools v1.14 (Danecek et al. 2021) to remove unmapped and mate-unmapped reads. PCR duplicates were identified and marked using MarkDuplicates in the PICARD package (https://broadinstitute.github.io/picard/). Genotype calling for each individual was performed using GATK HaplotypeCaller (https://github.com/broadinstitute/gatk/), and individual variant files were merged with CombineGVCFs. Variants were jointly genotyped with GATK GenotypeGVCFs to estimate parameters such as QUAL, allele count, and allele frequency. INDELs were excluded from subsequent analyses, resulting in 5,594,839 SNPs. Hard filtering was conducted based on the distribution of multiple statistics following GATK recommendations (QUAL < 40.0 || QD < 2.0 || MQ < 50.0 || FS > 30.0 || SOR > 8.0 || MQRankSum < −0.5 || ReadPosRankSum < −4.0), and loci unique to individual samples (max-missing 0.01) were removed, yielding 4,683,563 high-quality SNPs for all samples. Population structure analysis was conducted with ADMIXTURE v1.3.0 (Alexander et al. 2009), applying maximum-likelihood estimation of individual ancestries with *K* values ranging from 2 to 6 for all individuals of *A. baohuaensis*, *A. wanzhensis*, *A. hejiaqingii*, *A. yunmengensis*, *A. anhuiensis* and *A. yunjuensis* with 4,665,822 SNPs. For the admixture analysis, *K* was selected based on the resolution of species-level genetic clusters, with cross-validation (CV) values used as a reference rather than a strict criterion.

## Results

### Morphological comparison

**Figure 1.**
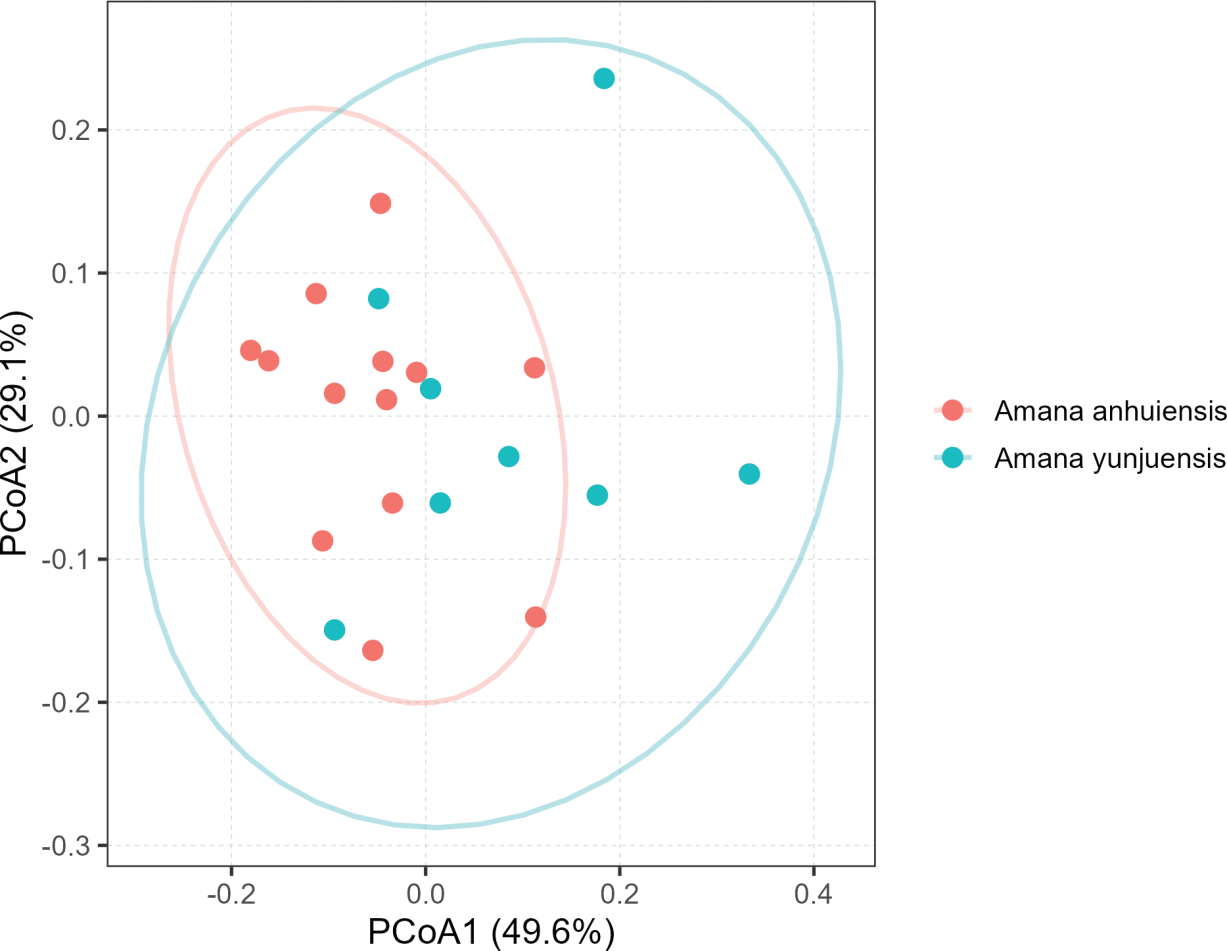
Principal coordinates analysis of *Amana anhuiensis* and *A. yunjuensis*. Ellipses represent the 95% confidence interval.

**Figure 2.**
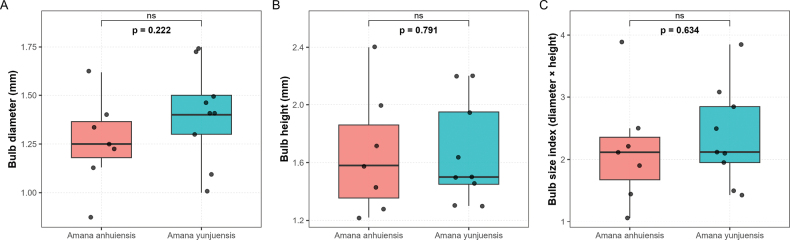
Comparison of bulb morphology between *Amana anhuiensis* and *A. yunjuensis*. Boxplots showing the variation in (A) bulb diameter, (B) bulb height, and (C) bulb size index (diameter × height) between *A. anhuiensis* and *A. yunjuensis*. Boxes indicate the interquartile range (IQR) and horizontal lines represent medians. The p-values above the plots were obtained from Wilcoxon rank-sum tests. No significant differences were detected for any of the three traits (*p* ≥ 0.05).

The results of principal coordinates analysis showed that *Amana yunjuensis* and *A. anhuiensis* were not morphologically distinguishable (Fig. 1). There was a clearly overlapping area between these two groups. The first principal component (PCoA1) and second principal component (PCoA2) accounted for 49.6% and 29.1% of total variance, respectively. Moreover, no significant morphological differences were detected in bulb traits between *Amana anhuiensis* and *A. yunjuensis* (Fig. 2). The two species exhibited largely overlapping variation ranges in bulb diameter (*p* = 0.222), bulb height (*p* = 0.791), and overall bulb size index (diameter × height; *p* = 0.634). Although *A. yunjuensis* tended to have slightly larger bulb diameter, this difference was not statistically significant (*p* ≥ 0.05). These results suggest that the bulbs of *A. anhuiensis* and *A. yunjuensis* are morphologically comparable, showing similar size and proportion despite minor individual variation.

### Phylogenetic analyses

**Figure 3.**
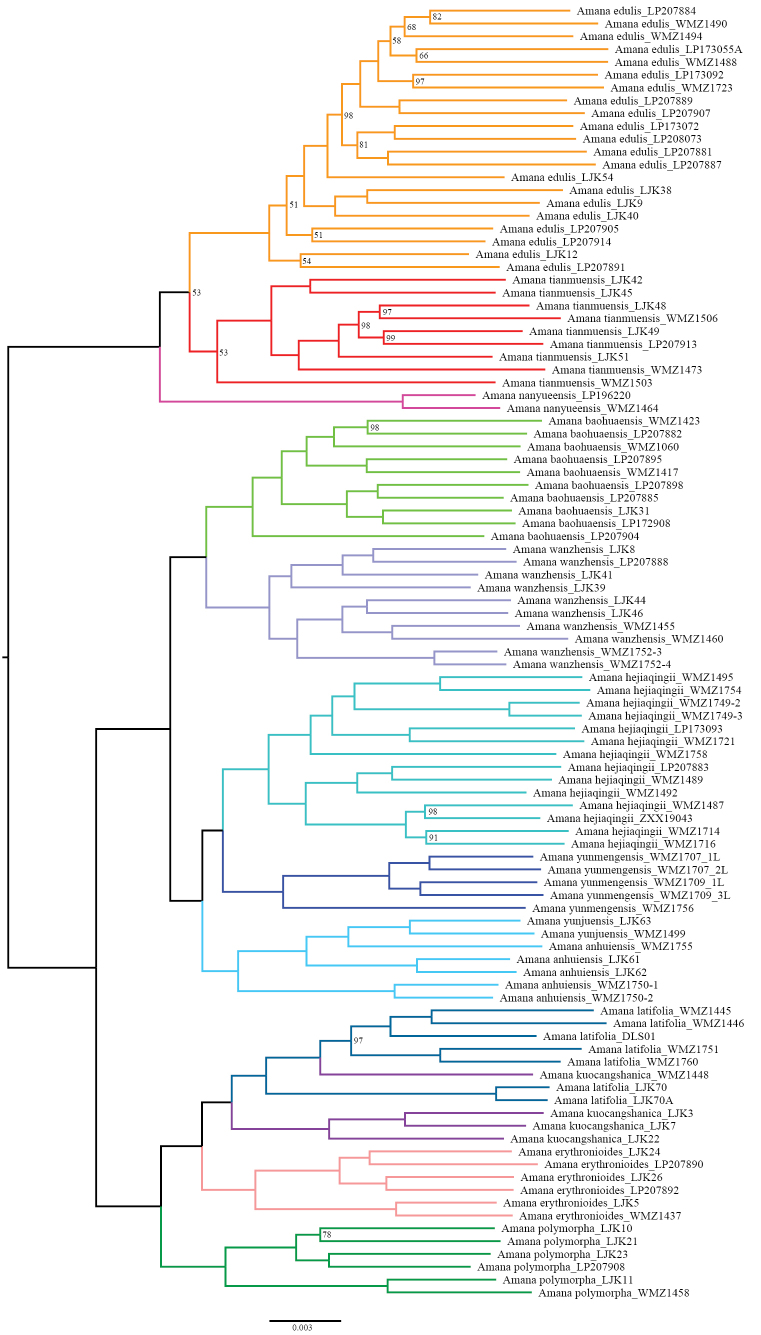
Phylogenetic tree of *Amana* based on nuclear genes. For visualization, the outgroup (*Erythronium japonicum*) was pruned from the displayed tree to avoid distortion caused by its deep divergence from the ingroup. All unlabelled nodes have 100% bootstrap support.

The phylogenetic tree based on nuclear genes revealed that *Amana yunjuensis* (LJK63, WMZ1499) was nested within the *A. anhuiensis* clade (WMZ1755, LJK61, LJK62, WMZ1750-1, WMZ1750-2), and the two species together formed a strongly supported monophyletic group (bootstrap = 100, Fig. 3). Besides, the phylogenetic relationship between *A. yunjuensis* and *A. anhuiensis*_WMZ1755 distributed in Mt. Lu in Jiangxi Province is the closest. Consistent with previous studies (Wang et al. 2024), *Amana* can be divided into three major clades: clade I ((*Amana edulis*, *A. tianmuensis*), *A. nanyueensis*), clade II ((*A. baohuaensis*, *A. wanzhensis*), ((*A. hejiaqingii*, *A. yunmengensis*), *A. anhuiensis*)) and clade III (*A. polymorpha*, (*A. erythronioides*, (*A. kuocangshanica*, *A. latifolia*))).

### Admixture analysis

**Figure 4.**
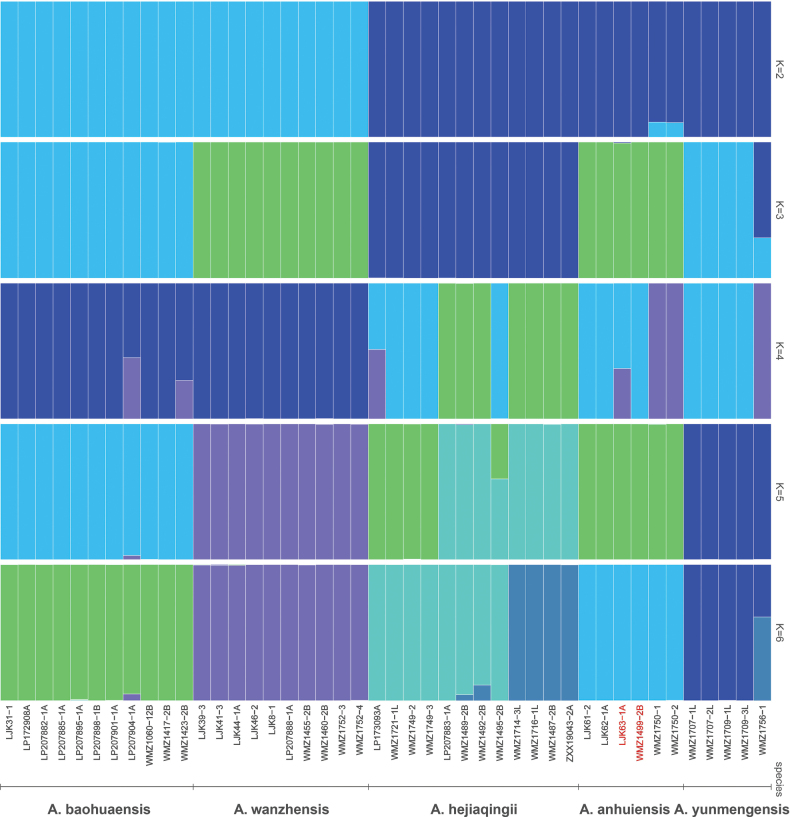
Admixture analysis result of clade II. The individual labelled in red is *Amana yunjuensis*.

The ADMIXTURE analysis (*K* = 2~6) clearly distinguished the species within clade II (*Amana baohuaensis*, *A. wanzhensis*, *A. hejiaqingii*, *A. yunmengensis*, *A. anhuiensis*). But the result showed that the two individuals of *A. yunjuensis* (LJK63 and WMZ1499) shared an identical ancestry composition with *A. anhuiensis* populations, without any unique genetic component across all *K* values (Fig. 4). This pattern, together with the phylogenetic nesting of *A. yunjuensis* within *A. anhuiensis*, indicates that these taxa are genetically indistinguishable and may represent recently diverged populations of the same species or conspecific lineages with ongoing or historical gene flow.

## Discussion

Species is the basic units of taxonomy, so reasonable species classification is necessary for the study of evolution and biology (Hong 2020). The integration of molecular evidence with classical morphological taxonomy is crucial for reliable species delimitation (Liu 2016). In this study, we combined morphological comparison, nuclear phylogenomics, and population genomic structure analysis to reassess the taxonomic status of *Amana yunjuensis*. The PCoA analysis based on morphological traits revealed substantial overlap between the two taxa, with no clear separation along the first two principal coordinate axes, which together explained 78.7% of the total variation. Likewise, the comparison of bulb traits, the diagnostic characters between these two species when published (Song et al. 2024), showed no statistically significant differences. Although *A. yunjuensis* exhibited slightly larger average bulb diameter, the variation ranges of the two taxa exhibited no significant difference, indicating that these differences likely reflect intraspecific variation rather than species level divergence.

Genomic evidence provided further support for their conspecific relationship. In the nuclear phylogeny, the two sampled individuals of *A. yunjuensis* were fully embedded within the *A. anhuiensis* clade, forming a strongly supported monophyletic lineage. Moreover, the individuals of *A. yunjuensis* showed the closest genetic relationship to *A. anhuiensis* populations collected from Mt. Lu in Jiangxi Province, near the type locality region of *A. yunjuensis*, suggesting geographic continuity rather than independent evolutionary history. Although, previous studies of *Amana* have shown that plastid-based phylogenetic trees do not recover individuals of *A. anhuiensis* and *A. yunjuensis* as a monophyletic clade. This pattern is likely attributable to extensive hybridization and introgression within the genus, potentially involving multiple maternal lineages, and represents a common feature of plastid phylogenetic analyses across *Amana* (Wang et al. 2024). Moreover, population structure analysis yielded fully concordant results: across all *K* values (*K* = 2~6), the two *A. yunjuensis* individuals displayed identical ancestry component to *A. anhuiensis* populations, without any detectable private genetic component. The absence of genetic differentiation, coupled with nuclear phylogenetic nesting and shared ancestry, strongly argues against the recognition of *A. yunjuensis* as an independent species.

Taken together, the lack of varying diagnostic morphological characters, complete phylogenomic embedding, and indistinguishable genomic composition collectively demonstrate that *Amana yunjuensis* does not represent a distinct evolutionary lineage but rather falls within the natural variation range of *A. anhuiensis*. Therefore, we conclude that *A. yunjuensis* should be treated as a taxonomic synonym of *A. anhuiensis*.

### Taxonomic treatment

#### Amana anhuiensis

Taxon classificationPlantaeLilialesLiliaceae

(X.S.Shen) D.Y.Tan & D.Y.Hong in Acta Bot. Boreal, 28: 0393-0395, 2008

C7A60792-3E95-515A-8E0A-AC4BFB29FBF8

##### Type.

China. • Anhui: Qianshan, Mt. Tianzhu. In bamboo forests or in bushes, 850~1,250 m alt., 14 March 2002, *D. Y. Tan & Z. Zhang* 皖*006* (neotype: PE; neoisotype: XJA).

#### = Amana yunjuensis

Taxon classificationPlantaeLilialesLiliaceae

B.X.Han & X.W.Song syn. nov.

D145D079-DCAE-5435-926D-EFA2AE80312A

##### Type.

China. • Jiangxi Province: Jiujiang City, Mt. Yunju, 5 Mar 2019, *Song SXW190305* (holotype: ACM; isotype: PE).

##### Description.

Perennial herbs; bulbs ovoid, 0.87–3.0 cm in diameter, tunics yellowish-brown, thinly papery, sparely villous-woolly inside. Stems 1.8–16.8 cm tall, glabrous, simple. Leaves usually 2, opposite, green with white veins, oblanceolate; the lower leaf 7.8–30.6 × 0.9–3.1 cm, the upper leaf 11–28.9 × 0.7–1.9 cm. Scapes 1.5–10.5 cm tall, glabrous, simple. Bracts usually 3, verticillate, narrowly lanceolate, green, 2–5 × 0.3–0.75 cm; pedicels 1.1–6.6 cm. Flowers solitary, funnel-shaped; tepals 6, white or light-pink, with a green or yellowish-green blotch at the very base inside and purple-red or light-pink streaks on the back; outer tepals lanceolate, acute, 2.3–4.5 × 0.6–0.95 cm, inner tepals elliptic-lanceolate, acute, 2.1–4.25 × 0.7–1.15 cm. Stamens 6, two-wheeled, the inner three slightly longer than the outer; filaments yellowish-green, 5–8.5(9) mm long, middle slightly dilated, gradually attenuate towards apex, glabrous; anthers light-purplish, the outer three 5–9.5 mm long, the inner three 6–9.5(10) mm long. Oval ovaries, yellowish-green, constricted below the style, 0.5–1 cm long, styles 0.4–0.7 cm long. Capsule subglobose, triquetrous, 0.5–1.31 cm in diameter, apex long beaked, 0.4–0.91 cm long. Fl. March–April, fr. April–May.

##### Phenology.

Flowering from March to April; fruiting from April to May.

##### Distribution and habitat.

*Amana anhuiensis* is distributed in Mt. Tianzhu in Anhui Province, Mt. Yunju and Mt. Lu in Jiangxi Province and Tuanfeng County in Hubei Province, China (Fig. 6). It mostly grows in moist deciduous broad-leaved forests and bamboo forests, at elevations of 600–1,710 m.

**Figure 5.**
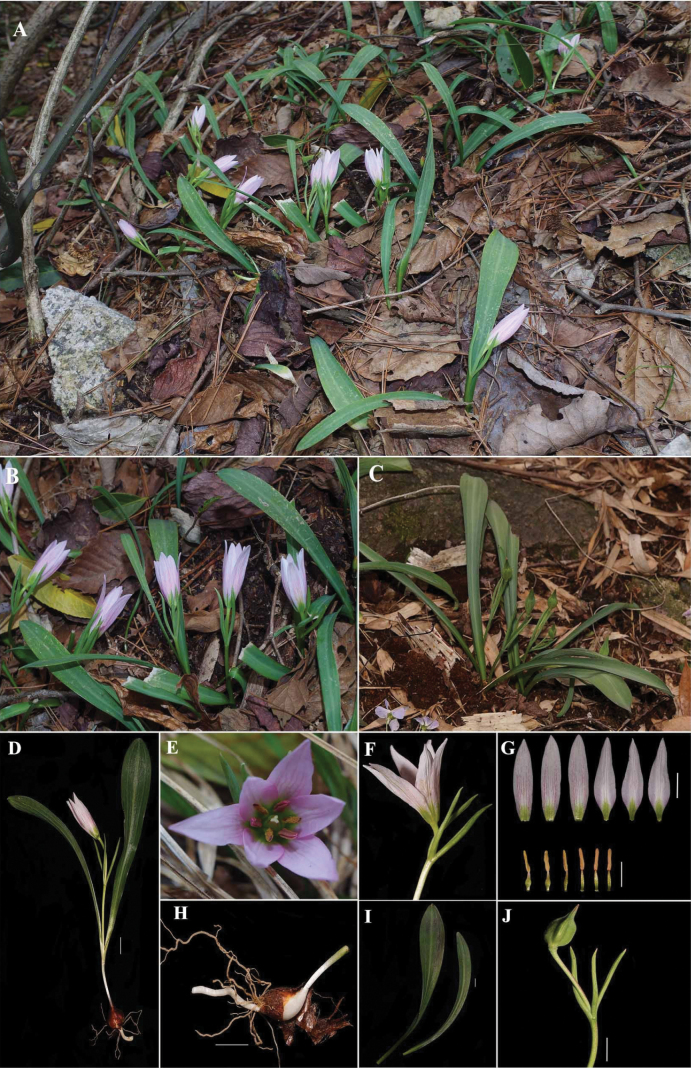
*Amana anhuiensis*. A. Habitat. B. Population with flowers. C. Population with fruits. D. Whole plant. E Front view of flower. F. Side view of flower. G. Floral dissection. H. Bulb. I. Leaves. J. Fruits. Scale bar = 1 cm. Photographs by Shenglu Zhang and Pan Li.

**Figure 6.**
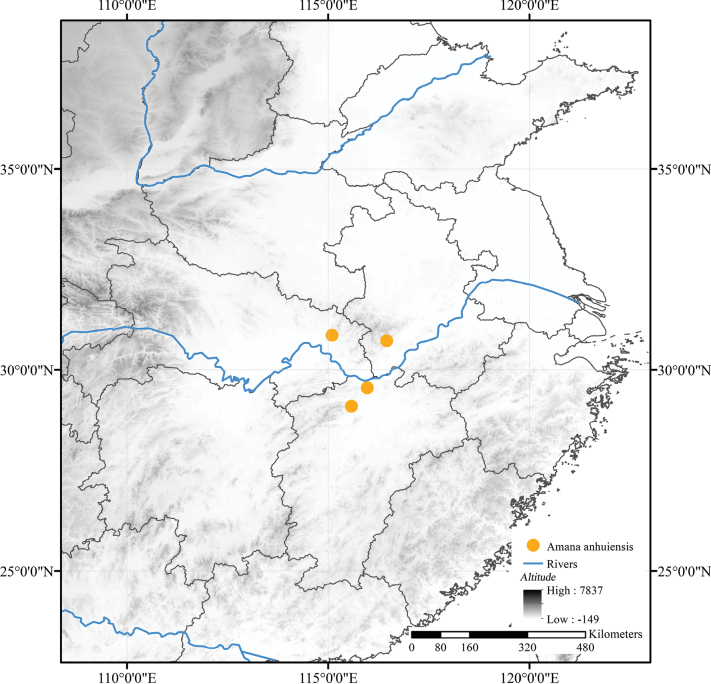
Distribution of *Amana anhuiensis*.

##### Additional specimens examined.

China. • **Anhui** Province: Qianshan County, Mt. Tianzhu, Zongguanzhai (总关寨), 850–1300 m, Fl. &Fr., 14 March 2002, *Dunyan Tan 006* (PE); *ibidem*, 1207 m, Fr., 9 May 2015, *Minqi Cai CMQ2015075* (HZU); *ibidem*, 1183 m, Fl., 4 April 2016, *Minqi Cai CMQ16215* (HZU); *ibidem*, 1183 m, Fl., 24 March 2019, *Pan Li & Junke Li LJK62* (HZU); Qianshan County, Mt. Tianzhu, Foguang Temple (佛光禅寺), 697 m, Fl., 4 April 2016, *Minqi Cai CMQ16216* (HZU); Qianshan County, Mt. Tianzhu (天柱山), 1711 m, Fl., 6 March 2021, *Pan Li et al. WMZ1480* (HZU). • **Jiangxi** Province: Jiujiang City, Yongxiu County, Mt. Yunju, Zhenru Temple (真如寺), 703 m, Fl., 21 March 2019, *Pan Li & Junke Li LJK 63* (HZU); *ibidem*, 711 m, Fl. & Fr., 10 March 2021, *Pan Li et al. WMZ1499* (HZU); Jiujiang City, Mt. Lu, Shimenjian (石门涧), 616 m, Fl., 11 March 2023, *Tongjun Liang LTJ 20230308* (LBG). • **Hubei** Province: Huanggang City, Tuanfeng County, Jiamiao Town, 789 m, Fl., 5 March 2023, *Xinxin Zhu WMZ1750* (HZU).

##### Conservation status.

*Amana anhuiensis* is only known from four places in Mt. Tianzhu, Mt. Lu, Mt. Yunju and Tuanfeng County, but with thousands of individuals at each site, thus we suspect that *Amana anhuiensis* could be categorized as Vulnerable (VU) according to IUCN criteria (IUCN 2022).

## Supplementary Material

XML Treatment for Amana anhuiensis

XML Treatment for Amana yunjuensis

## Additional information

### Use of AI

No use of AI was reported.

### Author ORCIDs

Meizhen Wang https://orcid.org/0000-0002-3794-465X

Shenlu Zhang https://orcid.org/0000-0001-8511-9913

Liang Guo https://orcid.org/0009-0005-0683-3542

Qing Ma https://orcid.org/0000-0002-2322-8847

Pan Li https://orcid.org/0000-0002-9407-7740

### Data availability

RNA-seq data were deposited at the National Genomics Data Center, China National Center for Bioinformation/Beijing Institute of Genomics under BioProject accession number PRJCA013174.

## Supplementary materials

Supplementary material 1 Morphological data used in the principal coordinates analysis for *Amana anhuiensis* and *A. yunjuensis* Data type xlsx https://binary.pensoft.net/file/1518218This dataset is made available under the Open Database License (http://opendatacommons.org/licenses/odbl/1.0/). The Open Database License (ODbL) is a license agreement intended to allow users to freely share, modify, and use this Dataset while maintaining this same freedom for others, provided that the original source and author(s) are credited. Meizhen Wang, Shenlu Zhang, Liang Guo, Qing Ma, Pan Li

Supplementary material 2 Bulb diameter and height measurements of *Amana anhuiensis* and *A. yunjuensis* Data type xlsx https://binary.pensoft.net/file/1518219This dataset is made available under the Open Database License (http://opendatacommons.org/licenses/odbl/1.0/). The Open Database License (ODbL) is a license agreement intended to allow users to freely share, modify, and use this Dataset while maintaining this same freedom for others, provided that the original source and author(s) are credited. Meizhen Wang, Shenlu Zhang, Liang Guo, Qing Ma, Pan Li

## References

[B1] Alexander DH, Novembre J, Lange K (2009) Fast model-based estimation of ancestry in unrelated individuals. Genome Research 19: 1655–1664. 10.1101/gr.094052.109 PMC2752134 19648217

[B2] Chen XQ, Mordak HV (2000) *Tulipa* Linnaeus. In: Wu ZY, Raven PH (Eds) Flora of China (Vol. 24). Science Press, Beijing & Missouri Botanical Garden Press, St. Louis, 123–126.

[B3] Danecek P, Bonfield JK, Liddle J, Marshall J, Ohan V, Pollard MO, Whitwham A, Keane T, McCarthy SA, Davies RM, Li H (2021) Twelve years of SAMtools and BCFtools. GigaScience 10: 1–4. 10.1093/gigascience/giab008 PMC7931819 33590861

[B4] de Queiroz K (2007) Species concepts and species delimitation. Systematic Biology 56(6): 879–886. 10.1080/10635150701701083 18027281

[B5] Emms DM, Kelly S (2019) OrthoFinder: Phylogenetic orthology inference for comparative genomics. Genome Biology 20: e238. 10.1186/s13059-019-1832-y PMC6857279 31727128

[B6] Grabherr MG, Haas BJ, Yassour M, Levin JZ, Thompson DA, Amit I, Adiconis X, Fan L, Raychowdhury R, Zeng Q, Chen Z, Mauceli E, Hacohen N, Gnirke A, Rhind N, di Palma F, Birren BW, Nusbaum C, Lindblad-Toh K, Friedman N, Regev A (2011) Trinity: Reconstructing a full-length transcriptome without a genome from RNA-Seq data. Nature Biotechnology 29: 644–652. 10.1038/nbt.1883 PMC3571712 21572440

[B7] Haas BJ, Papanicolaou A, Yassour M, Grabherr M, Blood PD, Bowden J, Couger MB, Eccles D, Li B, Lieber M, MacManes MD, Ott M, Orvis J, Pochet N, Strozzi F, Weeks N, Westerman R, William T, Dewey CN, Henschel R, LeDuc RD, Friedman N, Regev A (2013) De novo transcript sequence reconstruction from RNA-seq using the Trinity platform for reference generation and analysis. Nature Protocols 8: 1494–1512. 10.1038/nprot.2013.084 PMC3875132 23845962

[B8] Han B, Zhong K, Huang L (2014) *Amana wanzhensis* (Liliaceae), a new species from Anhui, China. Phytotaxa 177: 118–124. 10.11646/phytotaxa.177.2.3

[B9] Honda M (1935) *Amana* a new genus of Liliaceae. Bulletin of the Chemical Society of Japan 6: 19–21.

[B10] Hong DY (2020) Gen‐morph species concept—A new and integrative species concept for outbreeding organisms. Journal of Systematics and Evolution 58(5): 725–742. 10.1111/jse.12660

[B11] IUCN Standards and Petitions Committee (2022) Guidelines for using the IUCN red list categories and criteria, version 15. Prepared by the Standards and Petitions Committee. https://www.iucnredlist.org/documents/RedListGuidelines.pdf

[B12] Kalyaanamoorthy S, Minh BQ, Wong TKF, Haeseler AV, Jermiin LS (2017) ModelFinder: Fast model selection for accurate phylogenetic estimates. Nature Methods 14: 587–589. 10.1038/nmeth.4285 PMC5453245 28481363

[B13] Li H (2013) Aligning sequence reads, clone sequences and assembly contigs with BWA-MEM. arXiv: 1303.3997v1302. https://arxiv.org/abs/1303.3997

[B14] Li W, Godzik A (2006) Cd-hit: A fast program for clustering and comparing large sets of protein or nucleotide sequences. Bioinformatics 22: 1658–1659. 10.1093/bioinformatics/btl158 16731699

[B15] Liang TJ, Wang MZ, Gao S, Hu YN, Peng YS, Li P (2024) *Amana anhuiensis*, A Newly Recorded Species of Liliaceae from Lushan Mountain, Jiangxi Province. Jiangxi Science 42(4): 695–697.

[B16] Liu JQ (2016) “The integrative species concept” and “species on the speciation way”. Shengwu Duoyangxing 24(9): 1004–1008. 10.17520/biods.2016222

[B17] Minh BQ, Schmidt HA, Chernomor O, Schrempf D, Woodhams MD, von Haeseler A, Lanfear R (2020) IQ-TREE 2: New models and efficient methods for phylogenetic inference in the genomic era. Molecular Biology and Evolution 37: 1530–1534. 10.1093/molbev/msaa015 PMC7182206 32011700

[B18] Ohwi J, Kitagawa M (1992) New Flora of Japan. Shibundo, Tokyo, 1716 pp.

[B19] Oksanen J, Simpson G, Blanchet F, Kindt R, Legendre P, Minchin P, O’Hara R, Solymos P, Stevens M, Szoecs E, Wagner H, Barbour M, Bedward M, Bolker B, Borcard D, Borman T, Carvalho G, Chirico M, De Caceres M, Durand S, Evangelista H, FitzJohn R, Friendly M, Furneaux B, Hannigan G, Hill M, Lahti L, Martino C, McGlinn D, Ouellette M, Ribeiro Cunha E, Smith T, Stier A, Ter Braak C, Weedon J (2025) vegan: Community Ecology Package. R package version 2.8-0. https://vegandevs.github.io/vegan/

[B20] Pedersen TL (2024) patchwork: The Composer of Plots. R package version 1.2.0. https://cran.r-project.org/web/packages/patchwork/index.html

[B21] Shen XS (2001) A new species of *Tulipa* (Liliaceae) from China. Yunnan Zhi Wu Yan Jiu 23: 39–40.

[B22] Song XW, Li B, Wang W, Xu T, Shen XX, Han BX, Yi SY (2024) *Amana yunjuensis* (Liliaceae), a new species from Jiangxi, China. Phytotaxa 644(3): 229–235. 10.11646/phytotaxa.644.3.6

[B23] Stamatakis A (2014) RAxML version 8: A tool for phylogenetic analysis and post-analysis of large phylogenies. Bioinformatics 30: 1312–1313. 10.1093/bioinformatics/btu033 PMC3998144 24451623

[B24] Tan DY, Li XR, Hong DY (2007) *Amana kuocangshanica* (Liliaceae), a new species from south-east China. Botanical Journal of the Linnean Society 154: 435–442. 10.1111/j.1095-8339.2007.00660.x

[B25] Tan DY, Li XR, Hong DY (2008) Neotypification and Additional Description of *Amana anhuiensis* (X.S. Shen) D.Y.Tan & D.Y.Hong (Liliaceae) from Anhui, China. Acta Botanica Boreali-Occidentalia Sinica 28(2): 0393–0395.

[B26] Wang L, Xing Q, Lu GY, Lu X, Zhao Q, Song XW, Han BX (2019) *Amana baohuaensis* (Liliaceae), a new species from East China. Phytotaxa 427: 43–50. 10.11646/phytotaxa.427.1.5

[B27] Wang MZ, Zhang SL, Wu J, Zhu XX, Liu ZC, Lu GY, Li P (2022) *Amana hejiaqingii* (Liliaceae), a new species from the Dabie Mountains, China. Taxonomy 2: 279–290. 10.3390/taxonomy2030022

[B28] Wang MZ, Fan XK, Zhang YH, Wu J, Mao LM, Zhang SL, Cai MQ, Li MH, Zhu ZSC, Zhao MS, Liu LX, Cameron KM, Li P (2023a) Phylogenomics and integrative taxonomy reveal two new species of *Amana* (Liliaceae). Plant Diversity 45: 54–67. 10.1016/j.pld.2022.03.001 PMC9975474 36876315

[B29] Wang MZ, Wang ZY, Zhang SL, Wu J, Li P (2023b) *Amana yunmengensis* (Liliaceae), a new species from the Yunmeng Lakes region, China. Phytotaxa 629: 13–34. 10.11646/phytotaxa.629.1.2

[B30] Wang MZ, Wu J, Zhang SL, Mao LM, Ohi-Toma T, Takano A, Zhang YH, Cameron KM, Li P (2024) Species delimitation in *Amana* (Liliaceae): Transcriptomes battle with evolutionary complexity. Cladistics: The International Journal of the Willi Hennig Society 40: 135–156. 10.1111/cla.12565 37983640

[B31] Wickham H (2016) ggplot2: Elegant Graphics for Data Analysis. Springer-Verlag, New York. 10.1007/978-3-319-24277-4_9

[B32] Wilcoxon F (1945) Individual comparisons by ranking methods. In: Kotz S, Johnson NL (Eds) Breakthroughs in Statistics. Springer Series in Statistics. Springer, New York. 10.1007/978-1-4612-4380-9_16

[B33] Wilkins JS (2009) The Classical Era: Science by Division. Species: A History of the Idea. University of California Press, Oakland, 9–34. 10.1525/california/9780520260856.003.0001

[B34] Yang Y, Smith SA (2014) Orthology inference in nonmodel organisms using transcriptomes and low-coverage genomes: Improving accuracy and matrix occupancy for phylogenomics. Molecular Biology and Evolution 31: 3081–3092. 10.1093/molbev/msu245 PMC4209138 25158799

